# The role of magmatism in the thinning and breakup of the South China Sea continental margin: Special Topic: The South China Sea Ocean Drilling

**DOI:** 10.1093/nsr/nwz116

**Published:** 2019-08-13

**Authors:** Zhen Sun, Jian Lin, Ning Qiu, Zhimin Jian, PinXian Wang, Xiong Pang, Jinyun Zheng, Benduo Zhu

**Affiliations:** 1 CAS Key Laboratory of Ocean and Marginal Sea Geology, South China Sea Institute of Oceanology, Chinese Academy of Sciences, China; 2 Innovation Academy of South China Sea Ecology and Environmental Engineering, Chinese Academy of Sciences, China; 3 Department of Geology and Geophysics, Woods Hole Oceanographic Institution, USA; 4 School of Ocean and Earth Science, Tongji University, China; 5 East South China Sea Petroleum Research Institute, Shenzhen branch of China National Offshore Oil Corporation, China; 6 Guangzhou Marine Geological Survey, Ministry of Natural Resources, China

## INTRODUCTION

Magmatism plays a key role in the process of continental margin breakup and ocean formation. Even in the extremely magma-poor Iberia and Newfoundland margin, studies of field outcrops have shown that syn-rift magmatism had participated in rifting from a very early stage and contributed directly to the rifting process. The final transition from exhumed continental mantle to the ocean formation is also triggered by the accumulation and eruption of magma [1]. Therefore, Atlantic-type passive continental margins are classified into two end-members: magma-poor (non-volcanic) and magma-rich (volcanic). The differences between them lie in whether a large amount of intrusive and extrusive magmatism from the mantle plume/hotspot is involved in the syn-rift and breakup stages. A magma-rich margin [2] should include the following characteristics: (i) a high-velocity lower crust (HVLC) caused by syn-rift mafic magma underplating; (ii) continental crust intruded by abundant sills and dikes; (iii) a large volume of seaward-dipping reflectors (SDRs) caused by flood basalt eruption or tuffs. All other margins are classified as magma-poor margins.

**Figure 1:**
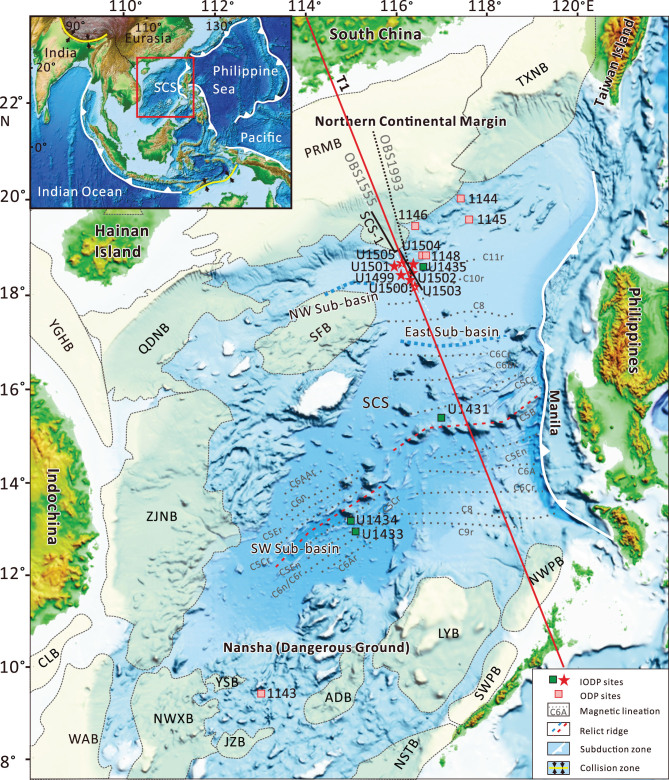
Geological setting of the South China Sea (SCS) as shown by a bathymetry map and ODP/IODP sites. The inset map on the upper left corner shows the subduction zones surrounding the SCS. The black line indicates the location of the seismic profile of Fig. 2; the red line indicates the position of the seismic tomography profile of Fig. 3. Sedimentary basins: PRMB, Pearl River Mouth basin; QDNB, Qiongdongnan basin; TXNB, Taixinan basin; YGHB, Yinggehai basin; ZJNB, Zhongjiannan basin; SFB, Shuangfeng basin; CLB, Cuu Long basin; WAB, Wanan basin; NWXB, Nanweixi basin; JZB, Jiuzhang basin; YSB, Yongshu basin; ADB, Andu basin; NSTB, Nansha Trough basin; LYB, Liyue basin; SWPB, Southwest Palawan basin; NWPB, Northwest Palawan basin.

The South China Sea (SCS) is a marginal sea surrounded by subduction zones on three sides (Fig. 1). Onshore outcrop and offshore drilling suggested that the SCS started rifting in Late Cretaceous following the Mesozoic subduction and developed into an ocean after early Oligocene. Although classified as a magma-poor margin, the northern continental margin of the SCS shows many features different from the classic examples of Iberia and Newfoundland, including the widely distributed (over 250 km) detachment faults, widespread HVLC and magmatic intrusion below extended crust, large amount of landward-dipping fault systems and especially the fast transition from continental margin-to-seafloor spreading [3]. Thus, the northern central SCS continental margin challenged the typical magma-poor and magma-rich models by having combined features of both.

**Figure 2:**
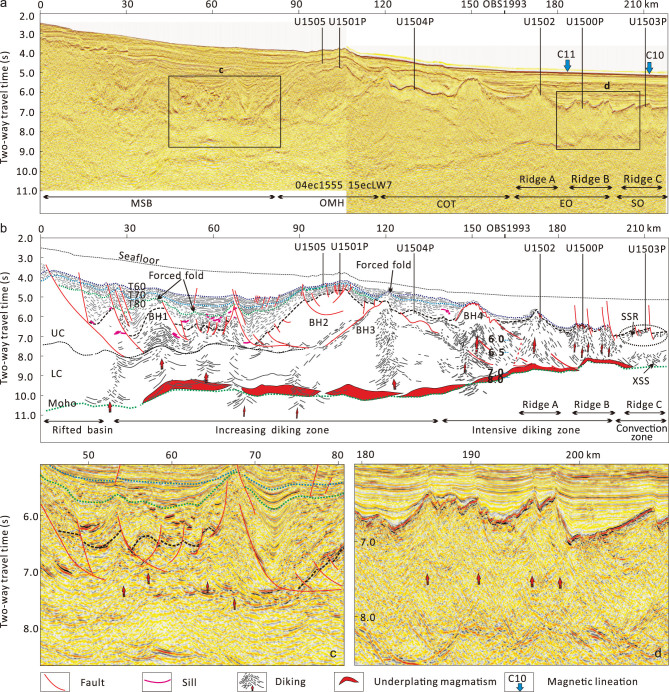
Original (a) and interpreted (b) transect SCS-1 across most of the drill sites. Zoom-in sections about diking are shown in (c) and (d). Interpretation highlights the sills, diking and underplating reflections, as well as the associated deformation of sedimentary sequences. MSB, mid-slope basin; OMH, Outer Margin High; COT, continent–ocean transition; EO, early ocean; SO, steady-state ocean; UC, upper crust; LC, lower crust; BH, basement high; XSS, X-shape shear structures; SSR, single-sided reflectors. IODP site names that end with a ‘P’ indicate sites that were not directly on the transect line but were parallel-projected. Magnetic lineation C11 has an age of about 30 Ma and C10 is about 28–29 Ma.

To test what kind of thinning process might have controlled the transition from continent to ocean in the SCS, International Ocean Discovery Program (IODP) Expeditions 367, 368 and 368X were carried out in 2017 and 2018. Seven sites were drilled from the Outer Margin High (OMH) to the continent–ocean transition (COT) and to the early ocean basin [4] (Figs 1 and 2). Neither serpentinized mantle nor flood basalt/tuff was encountered; instead, over 120 m fresh mid-ocean ridge basalt (MORB) with sediment interlayers were recovered at Site U1500 and ~180 m altered basalt at Site U1502. According to the magnetic anomaly interpretation, paleontological dating in sediment and ship-board major-element analysis, the earliest MORB is about 30–34 Ma [4]. How is the transition from continent to ocean fulfilled? Discriminating the magmatic process of the SCS continental margin will greatly help to understand its thinning and breakup processes.

### DIKING AND UNDERPLATING FEATURES

Constrained by the new IODP drilling data, seismic reflection and OBS refraction-velocity sections, we are able to determine first-order architecture of the syn-rift sequences, pre-rift basement, Moho and upper/lower-crust boundaries (Fig. 2). In the highly extended Liwan sag, where the crust has been thinned to 6–8 km, high-amplitude normal-polarity sill reflections are observed (at the profile distance of ~20–80 km). Similar reflections are also observed in the upper crust and close to the basement high at location BH4 (at profile distance of ~140 km). Increasing diking reflections are also observed from below the Liwan sag until Ridge B. Most of the diking reflections are consistent with the convexing upper/lower-crust boundary and can reach Tg or even shallower (Fig. 2). In the COT, diking is seen to penetrate through the detached wedge and reach the seafloor (Fig. 2), where altered basalt was recovered at Site U1502 [4]. Below Ridge B, where Site U1500 is projected, seismic reflection shows closely arrayed dikes (Fig. 2). According to analysis of the sediment stratigraphy, diking occurred in three episodes: around T80 (~40 Ma) [5] in the Liwan sag, around T70 (~30–34 Ma) in the OMH and COT (below locations BH3 and BH4 in Fig. 2) and about T60 (~24–28 Ma) in the early ocean basin (below Ridges A and B). Ridge C basement shows single-sided reflectors dipping landward. We therefore speculate that, below Ridge C, convection of the mantle has substituted the diking process and steady-state spreading has occurred. In sum, intrusive sills and diking may have occurred in the distal margin over a long distance of ~200 km. Meanwhile, syn-tectonic sedimentation deformation suggests that such dike intrusion has taken place from T80 (~40 Ma) to T60 (~24–28 Ma) and became younger oceanward.

**Figure 3:**
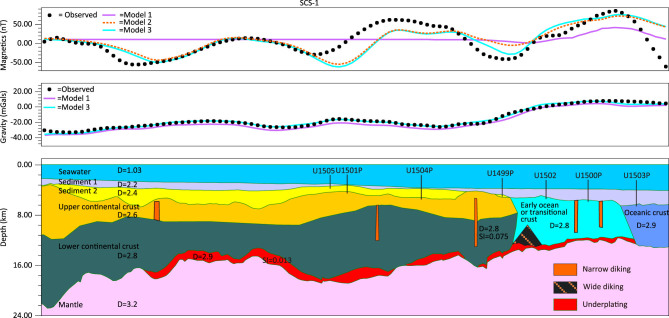
Forward modeling of transect SCS-1 in three scenarios. Model 1 has neither underplating nor diking; Model 2 has underplating but no diking; and Model 3 has both underplating and diking. Models 2 and 3 both provide adequate fits to the gravity and magnetic data. The contributions to the magnetic anomaly are from both continent/ocean crust and a magmatic underplating layer. Narrow dikes do not cause obvious magnetic anomalies.

Above the Moho, a layer of strong reflection is observed, thinner in the COT and thicker below the Liwan sag. The strong reflectors above the Moho correspond to HVLC in the crosspoint with seismic line OBS1993 (Fig. 2). We conjecture that the strong reflections may represent the magmatic underplating, which is not continuous across the margin and is sometimes obscured by the diking. Further seaward below Ridge C, where fresh basalts have been recovered at Site U1503, the Moho reflection is characterized by a thick belt (~500 ms) of X-shaped shear structures. The X-shaped reflections are usually considered to be caused by ductile shearing between the crust and mantle below the mature oceanic crust and may contain interstitial melts. Thus, before the breakup, magmatic underplating and diking might have initiated, with the dikes injected into the crust or sediments to form sills.

We used gravity-magnetic anomaly forward modeling based on reflection and refraction seismic data to validate the above model. The seismic line SCS-1 (Fig. 3) has the best reflection quality showing clear segment boundaries, relatively good velocity control from refraction data and the IODP drilling data. The low-amplitude magnetic anomaly in the continental margin is shown to be contributed by two sources if not considering the magnetic reversals. The underplating of magma is interpreted to be composed of gabbro and cumulate, and is responsible for the observed long-wavelength anomaly, while basement topography accounts for the smaller-scale anomalies. Modeling showed that the intruding dikes do not contribute significantly to magnetic anomaly unless the dikes are wide enough. The magnetic anomaly increases to over 80 nT in Ridge B and then drops to −60 nT in Ridge C without large basement topographic variations, so the observed variations are interpreted to be caused by geomagnetic reversal. Gravity anomaly shows a large gradual increase of over 20 mGals towards the early ocean basin, which might suggest a gradual replacement of the continental crust by mafic components.

**Figure 4:**
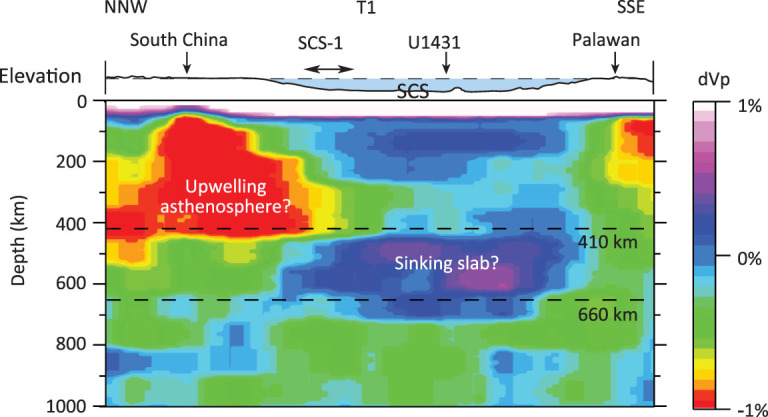
Seismic tomographic transect across the SCS from the MITP08 tomography model [9]. The lithosphere of the SCS overlaps partially with the low-velocity upwelling asthenosphere, suggesting that the northern part of the SCS might be affected by the upwelling. The horizontal black line with arrows on both ends shows the location of the seismic profile SCS-1.

**Figure 5:**
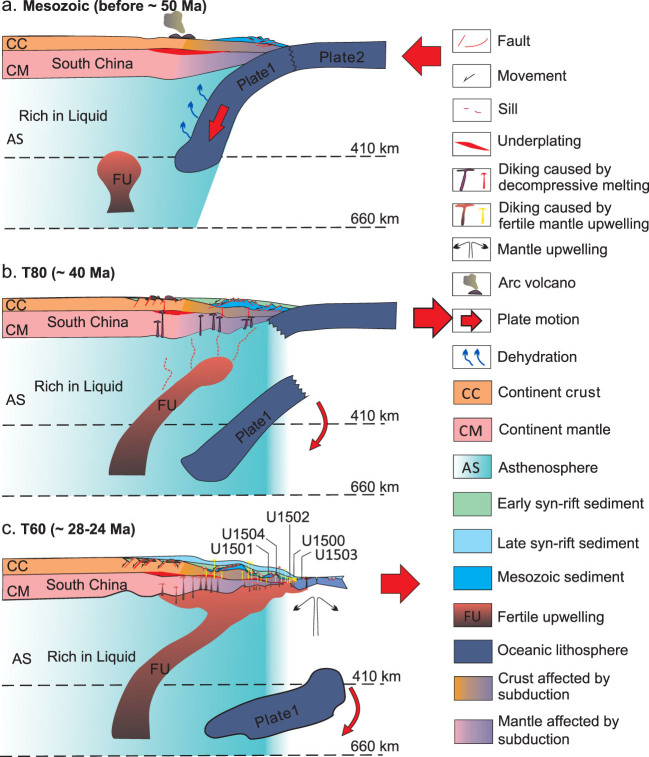
A conceptual mantle-scale model of the SCS showing the role of magmatic processes in rifting and breakup. (a) Mesozoic subduction made the lithosphere and asthenosphere below the South China more hydrated and fractured. (b) During the early rifting stage, extension led to increasing decompressive melting, which in turn led to increased diking and underplating towards the COT. (c) During the late-syn-rifting to early-spreading stages, fertile mantle upwelling may have provided more high-magnesium magma and led to the high-velocity lower-crust underplating, diking and eruption. Plates 1 and 2 might be the northern and southern slabs of the proto-South China Sea plate.

In summary, forward modeling of gravity and magnetic anomalies is consistent with the model of underplating in the distal margin of the SCS that contributes to the long-wavelength magnetic anomaly. Comparing with the magma-poor margins in the Atlantic, the SCS has more magma in its late-rifting-to-early-breakup stage, showing a combined feature of both magma-poor (lack of SDRs) and magma-rich (magmatic underplating).

### RIFTING AND BREAKUP MODEL OF SCS

Without SDRs being observed, most researchers suggested that the HVLC in the northern continental margin was underplated either in the Mesozoic pre-rift or post-rift stages. The widely distributed HVLC below the highly extended rift center and COT area discords with the pre-rift model. Yao [6] suggested that the HVLC should be underplated mainly during the syn-rift stage by upper-mantle melting. Comparison of the vertical P-wave velocity structure with other margins suggests that the HVLC in the highly extended COT area shows strong affinity to passive continental margin extension.

Evidence for the melting of a fertile asthenospheric mantle has been found in the middle Miocene E-MORB samples; it was also detected in the ocean island basalt-type samples from early to late Miocene, but not in the 25-Ma MORB samples recovered in southern Taiwan [7]. Thus it is proposed that this fertile asthenosphere upwelling might have arrived at the seafloor after ~25 Ma; this is consistent with the model of increasing magmatism during early Miocene in the NE continental margin, where drilling and dredging on the seamounts suggest a peak age of 22–24 Ma [8]. Other studies have proposed that such an upwelling of fertile mantle might be caused by a mantle plume after early Miocene [8]. However, the subsidence history of the Pearl River Mouth basin does not support models of plume-related strong uplift after early Miocene; instead, accelerated subsidence occurred thereafter and the slope area transitioned into a deep-water environment [5]. On seismic profiles, widespread uplift and erosion accompanied by diking or intrusion were observed before T60 and draping sequences thereafter (Fig. 2). Thus, we propose that the main magmatic upwelling and underplating may have occurred before early Miocene during the late-syn-rift to early-spreading stage (T80 to T60), reaching a maximum emplacement before T60. Similarly to many distal magma-rich margins, the mantle upwelling, magmatic underplating and eruption in SCS were controlled by extensional structures that are oriented in the NE to EW direction of the study area.

Major- and trace-element analyses suggest that the fertile composition of the upwelling asthenosphere showed strong signals of recycled plates, which might be originated from subduction [7]. Seismic tomography indicates that, beneath the SCS, there is a subducted slab lying in the mantle-transition zone. In front of this slab, a strong negative P-wave abnormal in the lithosphere may suggest mantle upwelling (Fig. 4) [9]. During rifting, the upwelling material may be dragged toward the thinning lithosphere and cause magmatic underplating, intrusion and eruption.

## CONCLUSIONS

Based on seismic interpretation, forward modeling of gravity and magnetics, seismic tomography and IODP drilling results, we suggest that the northern central margin of the SCS might have experienced two stages of magmatic process. It started with less magmatic extension and a highly extended margin with mainly seaward-dipping detachment faults formed. Diking induced by decompressive melting increased until breakup occurred around 30–34 Ma. Fertile upwelling arrived at the seafloor after ~25 Ma and led to magmatic underplating as well as seamount eruption. Given the co-existence of the MORB and Ocean Island Basalt, the two stages are not completely separated in time. The magmatic overlapping may play an important role in the late-rifting and especially spreading processes. Therefore, we propose that the SCS might represent a ‘plate-edge or Pacific-type’ extensional basin typical of marginal seas [10]. The structure and breakup process of such plate-edge extensional basins are not only controlled by stretching strains as in the case of the Atlantic-type ‘intra-plate’ extensional basins, but also affected strongly by surrounding subduction zones (Fig. 5). The Mesozoic subduction zones may have provided abundant fluids to the SCS continental margin, which make the crust and/or mantle very ductile during the syn-rift stage and rapid magma emplacement. Passive upwelling of the fertile asthenospheric mantle induced by the subduction zones may have provided abundant magma, including the deep-sourced high-magnesium magma, to the continental margin (Fig. 5b and c), leading to intrusion and underplating of mafic magma, formation of the HVLC and diking below the highly extended continental crust. Due to the passive upwelling, the northern SCS continental margin transitioned from a magma-poor to a magma-rich-like margin. Such magmatic processes might have occurred from the late-rifting to early-spreading stages, reaching maximum emplacement before T60 (~24–28 Ma). Magma eruption may have postdated the intrusion and underplating processes, causing post-rift or even post-spreading magmatism.

## Supplementary Material

NR_MS-2019-118_Supplementary_Data_nwz116
