# Supplementary material for: The role of magmatism in the thinning and breakup of the South China Sea continental margin: Special Topic: The South China Sea Ocean Drilling
Source: Natl Sci Rev. 2019 Aug 13;6(5):871–6. doi: 10.1093/nsr/nwz116 (PMC8291520; doi:10.1093/nsr/nwz116)
Supplement: NR_MS-2019-118_Supplementary_Data_nwz116 [file nr_ms-2019-118_supplementary_data_nwz116.docx]

**Supplementary materials**

**Abstract:**

IODP drilling indicates that the northern central South China Sea (SCS) continental margin challenged typical magma-poor and magma-rich models by showing features of both models. No flood basalt or tuff was recovered in the rifting-spreading sequences but widespread high velocity lower crust magmatic underplating and rapid transition from rifting to seafloor spreading. In order to reveal its special formation dynamics, the magmatic structure and activities were analyzed along the multi-channel seismic transect SCS-1 constrained with drilling and OBS data. We found that magmatism was very active during syn-rift stage from distal margin to the Continent Ocean Transition zone (COT). Sills and increased diking were observed across a long distance of about 200 km. Syn-tectonic sedimentation deformation suggested that diking happened from T80 (~40 Ma) to T60 (~24-28 Ma) and became younger oceanward. Increased diking finally led to seafloor spreading. Compared with magma-poor margins in Iberia-Newfoundland, South China Sea has more magma in its late-rifting to early breakup stage. Accelerated subsidence in the northern continental margin after early Miocene is inconsistent with the existence of an active mantle plume or hotspot. Therefore we propose that as a Pacific-type basin, the rifting and magmatic process of SCS might be controlled not only by stretching, but also by past subduction. Mesozoic subduction zones provided abundant fluid to the asthenosphere as well as the pre-existing structures to the upper crust, which may contribute to the wide zones of detachment faults, both inherited and newly formed. Passive upwelling of fertile asthenosphere caused by slab sinking may provide substantial magma and especially the deep sourced high magnesium magma, which in turn resulted in the underplating of HVLC and the associated diking below the highly extended crust.

1. **MORE ABOUT MAGMA-POOR AND MAGMA-RICH MARGIN**

Becker et al. (2014) analyzed 8 profiles along the magma-rich (volcanic) South Atlantic margin and found that high velocity lower crust (HVLC) locates not always below the seaward dipping reflectors (SDRs), and there is even one profile (No. 3) with HVLC being not accompanied with SDR. They suggested that HVLC can include two parts, one is horizontally distributed high velocity mafic magma and its cumulates, the other part is gabbroic intrusion toward extended continental crust. SDRs were caused by eruption of upwelling magmatism. Thus the SDRs may off-lap with HVLC due to many reasons, such as magma volume, pre-existing structures, asymmetric extension and so on. Only when magma increased to a certain degree, it can reach seafloor to cause flooding basalt eruption.

Geoffroy et al. (2015) proposed through mathematical modeling that magma-rich margin usually develops continentward-dipping normal faults (CDNF) due to active flow of ductile lithosphere, while magma-poor margin is mainly controlled by oceanward-dipping detachment faults (ODDF) due to dynamic necking. After comparing nine pairs of passive continental margins, Clerc et al. (2015, 2018) narrowed down the boundary between magma-poor and magma-rich margin unintentiously by including Angola-Gabon margins into magma-poor margins, where the faults dip mainly toward continent. They proposed that CDNFs are generated by faster flow/extension of the ductile lithosphere (lower crust and/or mantle) than the brittle upper crust, this process can happen when the lithosphere is warm or weak enough.

Based on above-mentioned research progress, the boundary between magma-poor and magma-rich margin became more and more obscure. The fault dipping direction cannot be regarded as one of the standards, and definition should mainly concerns about the magmatism. So magma-rich margin (Huismans et al., 2014; Geoffroy et al., 2015; Clerc et al., 2018) should include: (1) the high velocity lower crust (HVLC) caused by syn-rift mafic magma underplating; (2) continental crust strongly intruded by sills and dikes; (3) large volumes of Sea-ward Dipping Reflectors (SDRs) caused by flood basalt eruption or tuffs. And all the other types fall into the end of magma-poor margin.

Magmatic underplating may occur during rifting or spreading stages, but not necessarily with high velocity. For example, thick magmatic underplating without high velocity was deteced in Gulf of Carlifornia (Lizarralde et al., 2007; Han et al., 2016). Thus high velocity lower crust (HVLC) is usually assumed to be related with mantle plume/hot spot magmatic underplating due to high magnesium composition sourced from deep asthenosphere (White and McKenzie, 1989; Clerc et al., 2018).

1. **GEOLOGICAL BACKGROUND**

South China Sea (SCS) was a marginal sea surrounded by subduction zones on three sides (Figure 1). Onshore outcrop and offshore drilling suggested that SCS started rifting in late Cretaceous on the background of Mesozoic subduction zone (Li et al., 2018).

Rifting started in the Eocene (Pang et al., 2007) and led to seafloor spreading in late Oligocene (Briais et al., 1993). According to magnetic lineation interpretation, the oceanic crust formed early in the east around 30 Ma (Taylor and Hayes, 1983; Briais et al., 1993; Li et al., 2014) and propagated toward west. During extension, the northern continental margin was divided into several segments by NW-trending transfer faults. The highly extended continent-Ocean transition zone (COT) is wider in the east than in the west (Li et al., 2019). Inside the highly extended zone, NE-trending ridges with higher magnetic anomaly were observed ( Yeh et al., 2012). OBS velocity and joint gravity-magnetic forward modeling suggest a magmatic origin and three ridges were believed to be formed during early Miocene syn-rift or syn-spreading magmatism due to highly extension (Yeh et al., 2012; Sibuet et al., 2016). Drilling and dredging on the seamounts of the northern continental margin suggested a peak age of 22-24 Ma (Fan et al., 2017).

Our research area locates in the northern central margin, bordered through NW-trending Yitongansha Fault with the western margin. Joint seismic and gravity data analysis indicated that the extension generated two depocenters in the slope area, Baiyun and Liwan sags (Pang et al., 2007).

1. **DATA AND METHODS**

**3.1 SEISMIC DATA**

The multi-channel seismic data were acquired and processed by CNOOC in various surveys between 2004 and 2015. Relatively long streamers (6-7.5 km, 480-600 channels) and moderately-sized, tuned, airgun arrays (3680 to 4100 cu. in.) were used. Sample intervals are 1 or 2 ms, and broad recording bandwidth (open low-cut and up to 400 Hz high-cut filters) is utilized. The primary processing flow emphasized multiple attenuation and pre-stack time migration. The consistency in tilting angle between the sample measurement and the internal reflection of seismic profiles, either in sedimentary sequences or in basement, suggests that the oriented internal structure of the reflection seismic is reliable in identifying the geological deformation.

**3.2 FREE AIR GRAVITY ANOMALY DATA**

The free-air anomaly (FAA) of gravity data were taken from the global gravity model (V23) of Sandwell et al. (2014), which has a resolution of 30 arc-seconds, and an improvement by a factor of 2 to 4 relative to the old altimetry data (Geosat and ERS-1). Many structures can be discriminated from this new model, such as the Baiyun and Liwan depocenter and the Shenhu-Yitongansha Fracture zone (SHFZ).

**3.3 MAGNETIC ANOMALY DATA**

The total field magnetic intensity (TMI) anomaly data were taken using the one arc-minute dataset of East and Southeast Asia, which was compiled from available shipboard and some onland magnetic data by the Geological Survey of Japan and the Coordinating Committee for Coastal and Offshore Geoscience Programs in East and Southeast Asia (CCOP) (Ishihara and Kisimoto, 1996).

- 1. **JOINT FORWARD MODELING**

The joint forward modeling of the profiles has been constrained from nearby IODP sites as well as the oil and gas exploration drilling sites, reflection seismic profiles and velocity structures. Drilling results (Sun et al., 2018) suggest that the basement of the slope area are made of either Mesozoic sandstone or greenschist mylonite, which all show extremely weak magnetic susceptibility. According to drilling in the LF35-1-1 in neighbor areas and sites in the northern shelf area, the crystalized basement may also include Mesozoic granites (Chen et al., 2003), it also has weak magnetic anomaly. As gravity modeling, we give a reference to the trend of the physical properties of the crust based on the velocity conversion density. The parameters used in the forward modeling are listed in Table 1.

Table 1 Crustal seismic velocities and the corresponding density and magnetic susceptibility values obtained from the physical properties of near IODP site drilling and the NafeeDrake relationship for present study region (compiled from reference IODP and OBS).

| Layer | Continental crust | | | Transitional crust | | | Oceanic crust | | |
| --- | --- | --- | --- | --- | --- | --- | --- | --- | --- |
| Physical properties | Velocity range  (km/s) | Density  (g/cm3) | Magnetic Susceptibility  (10^-3^SI) | Velocity range  (km/s) | Density  (g/cm3) | Magnetic Susceptibility  (10^-3^SI) | Velocity range (km/s) | Density (g/cm3) | Magnetic Susceptibility  (10^-3^SI) |
| Postrift Sediments | 1.5~2.0 | 2.2 | 0.01~0.1 | 1.5~2.0 | 2.2 | 0.01~0.1 | 1.5~2.0 | 2.2 | 0.01~0.1 |
| Synrift Sediments | 2.0~4.0 | 2.4 | 0.01~0.1 | 2.0~4.0 | 2.4 | 0.01~0.1 | ~ | ~ | ~ |
| Upper crust | 5.5~6.2 | 2.6 |  | 5.5~6.2 | 2.8 |  | 6.0~8.0 | 2.9 | 1~10 |
| Lower crust | 6.8~6.9 | 2.8 |  | 6.8~6.9 | ~ |  | ~ | ~ | ~ |
| Dike | 6.8~6.9 | 2.8 | 10-70 | 6.8~6.9 | 2.8 | 10-70 | ~ | ~ | ~ |
| Magmatic Underplating | 7.2~8.0 | 2.9 | 10-20 | 7.2~8.0 | 2.9 | 10~20 | ~ | ~ | ~ |
| Upper mantle | 8.1~8.2 | 3.2 |  | 8.1~8.2 | 3.2 |  | 8.1~8.2 | 3.2 |  |

**4. RESULTS**

- 1. **SILLS AND DIKING**

Diking can be discerned on MCS seismic profiles for its through-going λ shape reflections in the crust and sediments (Figure S1, a-c). Strong sill reflections are often seen above or neighbor of the diking reflections. Sills are usually intrusive magma and distribute parallel to the sequences (Figure S1, b). Above the diking reflection, forced folds caused by magmatic upwelling can be observed. They can help dating the time of intrusions by dating the sediment or fossils immediately above the forced folds.


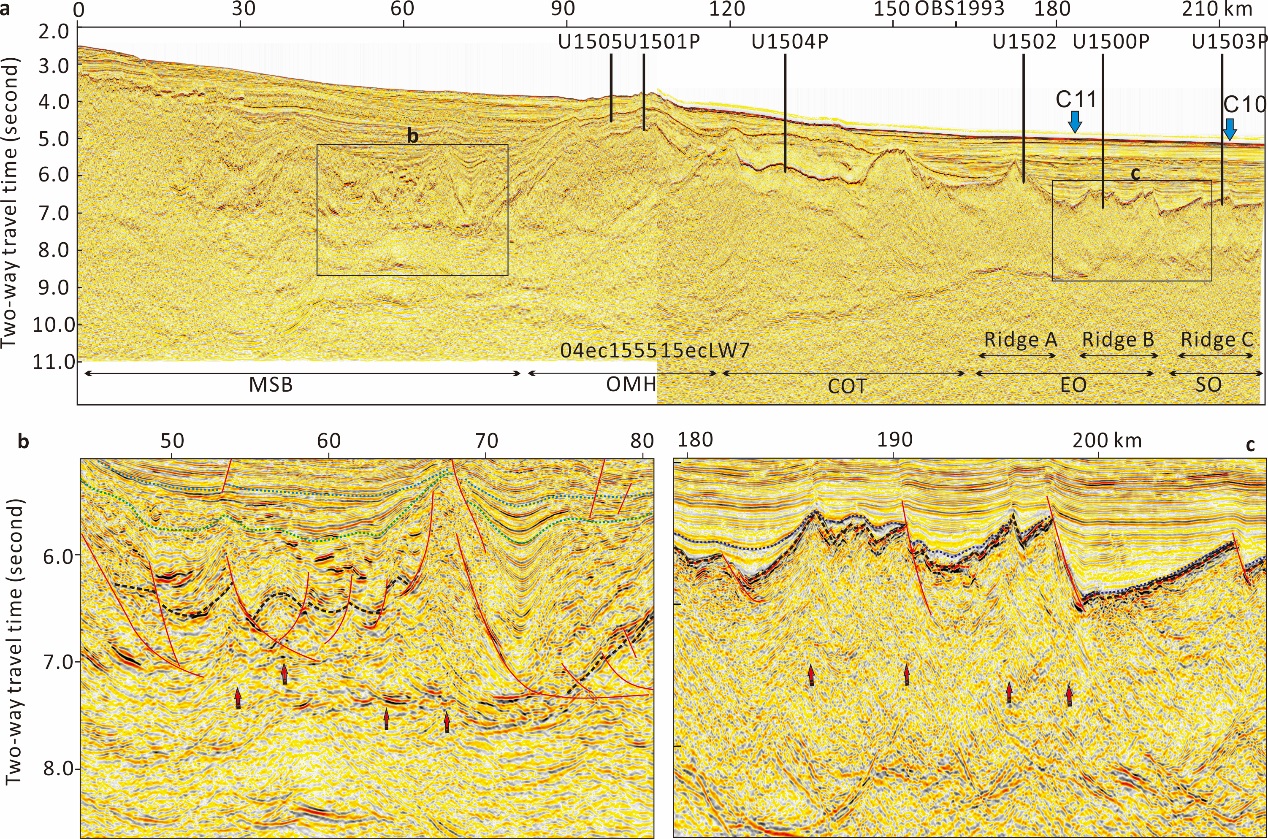


Figure S1 Two zoom-in seismic profiles (b, c) along the main transect (a) showing the densely distributed diking zone. b: The segment in the Liwan sag center; c. the segment in the early ocean area. The position of segement b and c was shown in a.

- 1. **FORWARD MODELING**

In order to test whether there is magma underplating and whether diking has caused anomaly on gravity and magnetic, we try to set up three different models in the comprehensive simulation, Model 1 has no magma underplating, Model 2 has magma underplating but no diking, and Model 3 has both underplating and diking with magnetic susceptibility (Figure S3).

The joint line SCS-1 (Figure S1) has the best reflection quality, involves clear segment boundaries, and has better control from refraction data (OBS 1993 of Yan et al., 2001 and a recent OBS1555, unpublished), as well as the IODP drill hole data (Sun et al., 2018). Comparison of three models (Figure 3) suggests that underplating is needed to simulate the gravity and magnetic anomaly. It contributes to the long wavelength magnetic anomaly of the continental crust, also slightly to the gravity anomaly. While diking contributes very little to either gravity or magnetic anomaly when it’s narrow, but will generate magnetic anomaly when it’s wide.

The low amplitude magnetic anomaly in the continental margin is contributed by two sources if not considering the magnetic reversals. The underplating of magma (composed of gabbro and cumulate according to Furlong and Fountain, 1986; White and McKenzie, 1989; Christensen and Mooney, 1995) is responsible for the long wavelength anomaly, while basement topography accounts for the superimposed smaller-scale anomalies. Intruding dikes didn’t show obvious magnetic anomaly unless they are wide enough. The magnetic anomaly increased to over 80 nT in Ridge B and then dropped to -60 nT in Ridge C without large basement togpography variation, so the variation was considered to be caused by geomagnetic reversal. The gravity anomaly shows a slow but large increase (over 20 mGals) in the early ocean segment, which might suggest a gradual replacement of the continental crust by mafic components.

In summary, forward modeling of gravity and magnetic anomaly verifies that underplating occurred in the distal margin of the SCS and contributes to long wavelength magnetic anomaly for the continental margin. Compared with magma-poor margins in Atlantic, South China Sea has more magma in its late-rifting to early breakup stage and shows a combined feature of both magma-poor and magma-rich (magmatic underplating).

- 1. **TOMOGRAPHIC EVIDENCES**

According to Seton et al. (2015) and Zhang and Li (2018)’s mathematical modeling, the South China Sea is located above an area surrounded by sinking subducting slabs, which will stimulate deep asthenosphere upwelling and generate mantle plumes as Hainan. While Maruyama et al. (2007) believes that surrounded by subduction, this area should have the coldest mantle, but hydrous plumes at 410 km depth might be easily formed due to the breakdown reaction of hydrous wadsleyite enriched in incompatible elements. In order to trace the deep sources for magmatic upwelling, we cut a tomographic transect across the SCS from global P-wave tomography model MITP08 (Li et al., 2008). The transect coincides with SCS-1 in the north from COT to early ocean area. On either side of the sinking slab, there is a cluster of low velocity material, which was supposed to be upwelling asthenosphere corresponding to the sinking of the slab. During rifting, the upwelling material may be dragged toward the thinning lithosphere and cause magmatic underplating, intrusions and eruptions.

**5. RIFTING AND BREAKUP MODEL OF SOUTH CHINA SEA**

Without SDRs being recognized, the SCS has been classified into magma-poor margin (Song et al., 1998; Franke et al., 2014; Clerc et al., 2018). So most scientists suggested that the HVLC in the east northern continental margin were underplated either in Mesozoic pre-rifting (Nissen et al., 1995) or during post-rifting stages (Zhao et al., 2010; Fan et al., 2017; Xia et al., 2018). Yao (1998) suggested that the HVLC should be underplated mainly during syn-rift stage by upper mantle melting. After comparing vertical velocity structure with other margins, Wan et al. (2017) suggested that the HVLC in Dongsha rise has the feature of volcanic arc and might be formed by Mesozoic subduction, while in highly extended COT area, the HVLC shows strong affinity to passive continental margin extension. They suggested it as syn-spreading underplating. The widely distributed HVLC below highly extended rift center and COT area discords with pre-rift model. Considering the syn-rift magmatic diking and force fold deformation (Figure 2) happened mainly from T80 to T60, we propose that the associated underplating should happen at similar age. This is consistent with the age group of magmatism at 40-22 Ma which were widely drilled in Pearl River Mouth basin (Chen et al., 2003) and discovered in the outcrop of Taiwan island (Huang et al., 2013; 2018).

Scientists discovered the melting of a fertile asthenosphere in the middle Miocene MORB samples (Zhang et al., 2018) as well as ocean island basalt-type samples from early to late Miocene, but not in the 25 Ma MORB found in Southern Taiwan (Yu et al., 2018). So Yu et al. (2018) conjectured that this fertile asthenosphere upwelling might arrive at surface after 23.8 Ma, which is consistent with the increasing magmatism during early Miocene in the NE continental margin, as well as the widespread uplift and erosion around T60. Some scientists conjectured this fertile upwelling was caused by plume during post-spreading stage (Fan et al., 2017; Xia et al., 2018). However, subsidence history of Pearl River Mouth basin doesn’t support a plume or hotspot related strong uplift after early Miocene, but accelerated subsidence (Xie et al., 2006; Zhao et al., 2011; Xie et al., 2014). So we propose that the main magmatic upwelling and underplating may happen during late syn-rift to early spreading stage. Similar to many far-field magma-rich margins, the mantle upwelling, magmatic underplating and erupting were controlled by extensional structures and might misfit each other (Becker et al., 2014). Fertile composition of the upwelling asthenosphere might come from the subduction of surrounding plates (Maruyama et al., 2007; Yu et al., 2017), especially the Mesozoic subduction beneath South China (Li et al., 2012; Li et al., 2018), like the Hainan plume (Zhang and Li, 2018).

**6. CONCLUSION**

Based on seismic interpretation and forward modeling of gravity and magnetics, we suggest that the northern central margin of SCS might have experienced two stages of magmatic process. It started with less magmatic extension and highly extended margin with mainly sea-ward dipping detachment faults formed. Diking induced by decompressive melting increased till breakup occurred around 30-34 Ma. Fertile upwelling arrived at the seafloor after ~25 Ma and led to magmatic underplating as well as seamount eruption. Given the co-existence of the MORB and OIB, the two stages are not completely separated in time. The magmatic overlapping may play an important role in late rifting and especially spreading process. Therefore, we propose that as a peri-plate basin, the rifting and magmatic process of SCS might represent a typical ‘Pacific-type’ extensional basin typical of marginal seas. The structure and breakup process of such extentional basins were controlled not only by stretching strain as the Atlantic type basins, but also by the fertile upwelling and pre-existing structures associated with subduction zones (Figure 5). Mesozoic subduction zone provided lots of fluid to the SCS continental margin, which make the crust and/or mantle very ductile during syn-rift stage and rapid magma emplacement (Figure 5a),and in turn may contribute to the wide zones of detachment faults (Pang et al., 2018), both inherited and newly formed. Fertile asthenosphere upwelling induced by subduction and plate sinking may provide lots of magma and especially the deep sourced high magnesium magma to the continental margin (Figure 5 b, c), which in turn led to the intrusion and underplating of mafic magma and the formation of HVLC as well as the strong diking below the highly extended crust during late rifting (T80, ~ 40 Ma) to early spreading stage (T60, ~24-28- Ma). Eruption may post date the intrusion and underplating process, and may last in post-rift stage and caused lots of post-rift or even post-spreading magmatism (Fan et al., 2017; Xia et al., 2018).

**REFERENCES:**

Becker, K.，Franke, D.，Trumbull, R.， Schnabel，M., Heyde， I., Schreckenberger，B., Koopmann, H., Bauer, K., Jokat, W., Krawczyk, C. M., 2014. Asymmetry of high-velocity lower crust on the South Atlantic rifted margins and implications for the interplay of magmatism and tectonics in continental breakup. Solid Earth, 5, 1011–1026.

Briais, A., P. Patriat, and P. Tapponnier, 1993. Updated interpretation of magnetic anomalies and seafloor spreading stages in the South China Sea: Implications for the Tertiary tectonics of Southeast Asia, Journal Geophysical Research, 98: 6299–6328.

Chen, C.-M., Shi, H.-S., Xu, S.-C., et al. 2003. The condition of oil and gas reservoir formation in the east of Zhujiangkou Basin. Scientific Publishing House, Beijing, China.

Childress, L., and the Expedition 368X Scientists, 2019. Expedition 368X Preliminary Report: South China Sea Rifted Margin.  International Ocean Discovery Program.  <https://doi.org/10.14379/iodp.pr.368X.2019>

Christensen, N. I, Mooney W. D. (1995) Seismic velocity structure and composition of the continental crust. J Geophs Res 100: 9761–9788

Clerc C., Jolivet L., Ringenbach J. C., 2015. Ductile extensional shear zones in the lower crust of a passive margin. Earth and Planetary Science Letters 431: 1–7.

Clerc, C., Ringenbach, J.-C., Jolivet, L., Ballard, J.-F., 2018. Rifted margins: Ductile deformation, boudinage, continentward-dipping normal faults and the role of the weak lower crust. Gondwana Research, 53 (2018): 20–40. http://dx.doi.org/10.1016/j.gr.2017.04.030

Ding, W.-W., Sun, Z., Dadd, K.,Fang, Y.-X., Li, J.-B., 2018. Structures within the oceanic crust of the central South China Sea basin and their implications for oceanic accretionary processes. Earth and Planetary Science Letters, 488 (2018) 115–125. <https://doi.org/10.1016/j.epsl.2018.02.011>

Fan, C., S. Xia, F. Zhao, J. Sun, J. Cao, H. Xu, and K. Wan (2017), New insights into the magmatism in the northern margin of the South China Sea: Spatial features and volume of intraplate seamounts, Geochem. Geophys. Geosyst., 18, 2216–2239, doi:10.1002/2016GC006792.

Franke, D., 2014. Rifting, lithosphere breakup and volcanism: Comparison of magma-poor and volcanic rifted margins, Mar. Petrol. Geol., 43, 63–87, 2013.

Furlong, K. P., Fountain, D. M., 1986. Continental Crustal Underplating: Thermal considerations and seismic-petrologic consequences, J. Geophys. Res., 91, 8285–8294, 1986.

Geoffroy, L., Burov, E.B., Werner, P., Volcanic passive margins: another way to break up continents. Sci. Rep. 5, 14828; doi: 10.1038/srep14828 (2015).

Han L., Hole J. A., Stock, J. M. et al., 2016. [Continental rupture and the creation of new crust in the Salton Trough rift, Southern California and northern Mexico: Results from the Salton Seismic Imaging Project](https://agupubs.onlinelibrary.wiley.com/doi/10.1002/2016JB013139). Journal of Geophysical Research-Solid Earth, 121(10) 7469–7489, doi:10.1002/2016JB013139.

Huang, C.-Y., Chi, W.-R., Yan, Y., Yang, K.-M., Liew, P.-M., Wu, M.-S., Wu, J.-C., Zhang, C., 2013. The first record of Eocene tuff in a Paleogene rift basin near Nantou, Western Foothills, central Taiwan. J Asian Earth Sci 69:3–16.

Huang, C.-Y., Shao, L., Wang, M.-H., Xue, W.-G., Qiao, P.-J., Cui, Y.-C., Hou, Y.-L., 2018. Benthic foraminiferal fauna and sediment provenance of Eocene synrift sequences in Taiwan: implication for onset of Asian epi-continental marginal seas off China coast. Marine Geophysical Research, https://doi.org/10.1007/s11001-018-9366-3

Huismans, R. S., Beaumount, C., 2014. Rifted continental margins: The case for depth-dependent extension. Earth and Planetary Science Letters, 407 (2014) 148–162.

Ishihara, T. and K. Kisimoto, 1996. Magnetic anomaly map of East Asia, scale 1:4.000.000 [CD-ROM], Geol. Surv. Of Jpn. and Coord. Comm. for Costal and Offshore Geosci. Programs in East and Southeast Asia, Tokyo, Japan.

Keating, P., Zerbo, L., 1996. An improved technique for reduction to the pole at low latitudes. Geophysics 61, 131–137.

Larsen, H. C., Mohn, G., Nirrengarten, M., et al., 2018. Rapid transition from continental breakup to igneous oceanic crust in the South China Sea. Nature Geoscience, <https://doi.org/10.1038/s41561-018-0198-1>.

Lizarralde, D., Axen G.J., Brown, H. E., et al., 2007. Variation in styles of rifting in the Gulf of California

Li C., van der Hilst and Engdahl RD et al. A new global model for P wave speed variations in Earth's mantle: Geochemistry, Geophysics, Geosystems 2008, 9(5): Q05018.

Li, C.-F., Xu, X., Lin, J., Sun, Z., Zhu, J., Yao, Y., Zhao, X., Liu, Q., Kulhanek, D.K., Wang, J., Song, T., Zhao, J., Qiu, N., Guan, Y., Zhou, Z., Williams, T., Bao, R., Briais, A., Brown, E.A., Chen, Y., Clift, P.D., Colwell, F.S., Dadd, K.A., Ding, W., Almeida, I.H., Huang, X.-L., Hyun, S., Jiang, T., Koppers, A.A.P., Li, Q., Liu, C., Liu, Z., Nagai, R.H., Peleo-Alampay, A., Su, X., Tejada, M.L.G., Trinh, H.S., Yeh, Y.-C., Zhang, C., Zhang, F., Zhang, G.-L., 2014. Ages and magnetic structures of the South China Sea constrained by deep tow magnetic surveys and IODP Expedition 349. Geochemistry, Geophysics, Geosystems 15: 4958–4983. doi:10.1002/2014GC005567

Li, F.-C., Sun, Z., Yang H.-F., 2018. Possible spatial distribution of the Mesozoic volcanic arc in the present-day South China Sea continental margin and its tectonic implications. Journal of Geophysical Research: Solid Earth, 123, 6215–6235. <https://doi.org/10.1029/2017JB014861>

Li, F., Sun, Z., Pang, X., et al., 2019. Low-viscosity crustal layer controls the crustal architecture and thermal distribution at hyper-extended margins: Modeling insight and application to the northern South China Sea margin. Geochemistry, Geophysics, Geosystems. doi: 10.1029/2019GC008200.

Li X. Magnetic reduction-to-the-pole at low latitudes: Observations and considerations[J]. Leading Edge, 2012, 27(8):990-1002.

Maruyama, S., Santosh, S., Zhao, D., 2007. Superplume, supercontinent, and post-perovskite: Mantle dynamics and anti-plate tectonics on the Core–Mantle Boundary. Gondwana Research 11 (2007) 7–37. doi:10.1016/j.gr.2006.06.003

Nissen, S. S., Hayes, D. E., Buhl, P., Diebold, J., Yao, B. C., Zeng, W. J., Chen, Y., 1995. Deep penetration seismic soundings across the northern continental margin of the South China Sea. Journal Geophysical Ressearch, 100: 22407-22433.

Pang, X., Chen, C. M., Peng, D. J., et al., 2007. The Pearl River Deep-water Fan System & Petroleum in South China Sea (in Chinese). Beijing: Science Press.

Pang, X., Ren, J.-Y., Zheng, J.-Y., Liu, J., Yu, P., Liu, B.-J., 2018. Petroleum geology controlled by extensive detachment thinning of continental margin crust: A case study of Baiyun sag in the deep-water area of northern South China Sea. Petrol. Explor. Develop., 2018, 45(1): 29–42.

Ren, J. Y., Pang, X., Lei, C., et al., 2015. Ocean and continental transition in passive continental margins and analysis of lithospheric extension and breakup process: Implication for research of the deepwater basins in the continental margins of South China Sea. Earth Science Frontiers, 22: 102-114.

Sandwell, D. T., R. D. Muller, W. H. F. Smith, E. Garcia and R. Francis (2014), New global marine gravity model from CryoSat-2 and Jason-1 reveals buried tectonic structure, Science, 346: 65-67.

Schouten H, Mccamy K. Filtering marine magnetic anomalies[J]. Journal of Geophysical Research, 1972, 77(35):7089-7099.

Sibuet, J.-C., Yeh, Y.-C., Lee, C. -S., 2016. Geodynamics of the South China Sea, Tectonophysics, s 692 (2016) 98–119. <http://dx.doi.org/10.1016/j.tecto.2016.02.022>.

Song, H. B., Hao, T. Y., Jiang, W. W., 1998. The type and formation mechanism of the northern continental margin. Cundan Monograph, Beijing, Science Press:74-81.

Sun, Z., Stock, J., Jian, Z., McIntosh, K., Alvarez-Zarikian, C.A., and Klaus, A., 2016. Expedition 367/368 Scientific Prospectus: South China Sea Rifted Margin. International Ocean Discovery Program. <http://dx.doi.org/10.14379/iodp.sp.367368.2016>.

Sun, Z., Jian, Z., Stock, J.M., Larsen, H.C., Klaus, A., Alvarez Zarikian, C.A., and the Expedition 367/368 Scientists, 2018. South China Sea Rifted Mar­gin. Proceedings of the International Ocean Discovery Program, 367/368: College Station, TX (International Ocean Discovery Program). <https://doi.org/10.14379/iodp.proc.367368.2018>

Sutra, E., Manatschal, G., Mohn, G., Unternehr, P., 2013. Quantification and restora-tion of extensional deformation along the Western Iberia and Newfoundland rifted margins. Geochem. Geophys. Geosyst.14, 2575–2597. <http://dx.doi.org/10.1002/ggge.20135>.

Taylor, B., and Hayes, D. E., 1980. The tectonic evolution of the South China Sea basin. Hayes D E, ed., The tectonic and Geological Evolution of Southeast Asian Seas and islands, AGU, Geophysical Monograph, 23: 89-104.

Wan, K., S. Xia, J. Cao, J. Sun, and H. Xu, 2017, Deep seismic structure of the northeastern South China Sea: Origin of a high-velocity layer in the lower crust, J. Geophys. Res. Solid Earth, 122, 2831–2858, doi:10.1002/2016JB013481.

White, R., McKenzie, D., 1989. Magmatism at rift zones: the generation of volcanic continental margins and flood basalts. Journal of Geophysical Research 94, 7685-7729.

Wu, J., Suppe, J., 2018. Proto-South China Sea Plate Tectonics Using Subducted Slab Constraints from Tomography. Journal of Earth Science, 29(6): 1304–1318. https://doi.org/10.1007/s12583-017-0813-x

Xia, S.-H., Zhao, F., Zhao, D.-P., Fan, C.-Y., Wu, S.-G., Mi, L.-J., Sun, J.-L., Cao, J.-H., Wan, K.-Y., 2018. Crustal plumbing system of post-rift magmatism in the northern margin of South China Sea: New insights from integrated seismology. Tectonophysics, 744(2018): 227-238. <https://doi.org/10.1016/j.tecto.2018.07.002>

Xie, H., Zhou, D., Li, Y.-P., Pang, X., Li, P.-C., Chen, G.-H., Li, F.-C., Gao, J.-Y., 2014. Cenozoic tectonic subsidence in deepwater sags in the Pearl River Mouth Basin, northern South China Sea. Tectonophysics, 615–616 (2014): 182–198, <http://dx.doi.org/10.1016/j.tecto.2014.01.010>

Xie, X.-N., Müller, R. D., Li, S.-T., Gong, Z.-S., Steinberger, B., 2006. Origin of anomalous subsidence along the Northern South China Sea margin and its relationship to dynamic topography. Marine and Petroleum Geology 23 (2006) 745–765, doi:10.1016/j.marpetgeo.2006.03.004

Yan, P., Zhou, D., Liu, Z. S., 2001. A crustal structure profile across the northern continental margin of the South China Sea. Tectonophysics, 338:1-21.

Yao, B.-C., 1998. Crust structure of the northern margin of the South China Sea and its tectonic significance. Marine Geology and Quaternary Geology, 18(2): 1-16.

Yeh Y C, Hsu SK and Doo WB et al. Crustal features of the northeastern South China Sea: insights from seismic and magnetic interpretations. Mar Geophys Res 2012; 33:307–326.

Yu, M., Yan, Y., Huang, C.-Y., Zhang, X., Tian, Z., Chen, W.-H., & Santosh, M. (2018). Opening of the South China Sea and upwelling of the Hainan plume. Geophysical Research Letters, 45,2600–2609. <https://doi.org/10.1002/2017GL076872>

Yu, Y., Gao, S. S., Liu, K. H., Yang, T., Xue, M., Phon Le, K., 2017. Mantle transition zone discontinuities beneath the Indochina Peninsula: Implications for slab subduction and mantle upwelling. Geophys. Res. Lett., 44, 7159–7167, doi:10.1002/2017GL073528.

Zhao, M., Qiu, X., Xia, S., et al. 2010. Seismic structure in the northeastern South China Sea: S-wave velocity and Vp/Vs ratios derived from three-component OBS data. Tectonophysics, 480: 183–197. doi:10.1016/j.tecto.2009.10.004.

Zhang, G.-L., Luo, Q., Zhao, J., Jackson, M. G., Guo, L.-S., Zhong, L.-F., 2018. Geochemical nature of sub-ridge mantle and opening dynamics of the South China Sea. Earth and Planetary Science Letters 489 (2018) 145–155. https://doi.org/10.1016/j.epsl.2018.02.040

Zhao, Z.-X., Sun, Z., Xie, H., et al., 2011. Baiyun deepwater Cenozoic subsidence and lithospheric stretching deformation. 54(12): 3336-3343. Doi: 10.3969/J.issn.0001 5733.2011.12.03.

Zhang, N., Li, Z.-X., 2018. Formation of mantle “lone plumes” in the global downwelling zone —A multiscale modelling of subduction-controlled plume generation beneath the South China Sea. Tectonophysics, 723 (2018) 1–13. <https://doi.org/10.1016/j.tecto.2017.11.038>.

Seton, M., Flament, N., Whittaker, J., Müller, R. D., Gurnis, M., Bower, D. J., 2015. Ridge subduction sparked reorganization of the Pacific plate-mantle system 60–50 million years ago. Geophys. Res. Lett., 42, 1732–1740, doi:10.1002/ 2015GL063057.
